# Governance of research involving people with psychosocial disabilities

**DOI:** 10.12688/wellcomeopenres.19279.1

**Published:** 2023-06-02

**Authors:** Marisha Wickremsinhe, Sarah Carrecedo, Aminu Yakubu, Aimi Nadia Mohd Yusof, Sharon Kaur

**Affiliations:** 1 London School of Hygiene and Tropical Medicine, London, UK; 2 Pontificia Universidad Catolica del Peru, Lima District, Lima Region, Peru; 3 Centre for Bioethics and Research, Ibadan, Nigeria; 4 University of Ibadan, Ibadan, Oyo, Nigeria; 5 Universiti Teknologi MARA, Shah Alam, Selangor, Malaysia; 6 Universiti Malaya, Kuala Lumpur, Federal Territory of Kuala Lumpur, Malaysia

**Keywords:** research ethics, mental health, psychosocial disability, research governance

## Abstract

Health-related research with human participants is governed by research ethics regulations in most jurisdictions. Globally, the 2016 International Ethical Guidelines for Health-related Research Involving Humans, published by the Council for International Organizations of Medical Sciences (CIOMS), are especially influential and widely held as an international standard. The CIOMS guidelines support the inclusion of people with psychosocial disabilities in research and offer clear guidance to promote their recruitment, including by outlining provisions for substitute decision-making. The CIOMS guidelines sit alongside the United Nations’ Convention on the Rights of Persons with Disabilities (CRPD). Adopted in 2006 and ratified in 2008, the CRPD offers a robust framework for recognizing the rights of persons with disabilities, including individuals with psychosocial disabilities. Though the CRPD does not explicitly reference research inclusion, its core principles—especially pertaining to the right to universal legal capacity—have clear implications for research ethics governance, specifically with respect to the use of substitute decision-making for research participation. In this paper, we review the extent to which existing research ethics regulations across selected jurisdictions concord with each of these two frameworks, offering first a broad analysis of regulations across 26 African countries, and then exploring two country-specific case studies from Malaysia and Peru. We find that, while many countries’ research ethics regulations align with key aspects of the CIOMS guidelines, core principles of the CRPD are absent. Given the shortcomings of existing regulations, we analyse a key point of tension between CIOMS and the CRPD—the right to participate in research—and offer a proposal for revised regulations that aims to bridge this tension and meet the standards of both frameworks.

## Disclaimer

The views expressed in this article are those of the authors. Publication in Wellcome Open Research does not imply endorsement by Wellcome.

## Introduction

The 2016 International Ethical Guidelines for Health-related Research Involving Humans, published by the Council for International Organizations of Medical Sciences (CIOMS), address the ethics of including people with psychosocial disabilities in research. Broadly, the CIOMS guidelines support highly inclusive target populations across studies to ensure fair distribution of research benefits. As such, the guidelines set out clear recommendations for the inclusion of people with psychosocial disabilities in research, with special attention to key issues related to capacity to consent to research and substitute decision-making. The CIOMS guidelines make clear that diagnoses of ‘mental and behavioural disorders’ do not necessarily imply incapacity to consent, marking progress on prior (and mistaken) views that having a psychosocial disability renders an individual incapable of giving informed consent as a matter of course ( CIOMS, 2016). Though the CIOMS guidelines move away from classifying groups of populations as inherently vulnerable, recognizing the harm that this designation has caused (in both sanctioning unfair research exclusion and perpetuating stigma toward specific sub-populations), the CIOMS guidelines do suggest that ‘some psychiatric conditions’ can cause incapacity, in which case surrogate decision-making is permitted ( CIOMS, 2016).

Conversely, a key aspect of the Convention on the Rights of Persons with Disabilities (CRPD) **—**and, indeed, one of its most celebrated and paradigm-shifting provisions—is its guarantee of the right to universal legal capacity for all individuals living with disabilities, including psychosocial disabilities ( UN, 2008). Though this right to universal legal capacity has been much debated in the literature ( *e.g.*, Appelbaum, 2019; Freeman *et al.*, 2015; Scholten & Gather, 2018) the Committee on the Rights of Persons with Disabilities reaffirmed in their General Comment No. 1 that legal capacity is, in fact, an absolute right that must be afforded to every individual, and that substitute decision-making of any kind contravenes the CRPD ( UN, 2014). Additional protections afforded in the CRPD include the right to equality, non-discrimination on the basis of disability, the right to full participation in society, and the right to the highest attainable standard of health. Though research participation is not explicitly mentioned in the CRPD, each of these provisions bear critical implications for the research enterprise.

While the CRPD can be understood as empowering individuals living with psychosocial disabilities, the CIOMS guidelines, while progressive, retain a key function of safeguarding individuals living with psychosocial disabilities. Two key tensions arise: firstly, while the CRPD prohibits substitute decision-making of any kind, the CIOMS guidelines permit substitute decision-making where the potential participant is deemed incapable of giving informed consent. Secondly, while the CRPD does not explicitly guarantee a right to participate in research, several of its core principles, especially in light of the CRPD’s guarantee to the right to health and the right to full participation in society, warrant further consideration and scrutiny in their application to the design and conduct of health research.

In this paper, we sought to first characterise the nature of a sample of existing research ethics regulations, across diverse jurisdictions, and the extent to which their mandates align with the CIOMS guidelines and the principles of the CRPD. First, we characterise existing regulations regionally, focusing on African countries. Then we offer in-depth reviews of two country-specific case studies: Malaysia’s Mental Health Act 2001 and Peru’s Regulation on Clinical Trials. Based on our analysis, we offer an orienting framework for revision of existing national research ethics regulations, aimed to align with *both* the CIOMS guidelines and the CRPD, delineated according to the purpose of the given research study. This paper was developed from presentations delivered during the ‘Governance of research involving people with mental health conditions’ session of the 2021 Global Forum on Bioethics in Research on ethical issues arising in research with people with mental health conditions.

## Review of selected regulations

### Regional study: Africa

Of 54 African countries, only 26 countries were reported to have guidelines regulating research (clinical and non-clinical) per the Office of Human Research Protections ( OHRP, 2019). Guidelines from 14 of these 26 countries (Botswana, Ethiopia, Ghana, Kenya, Liberia, Malawi, Nigeria, Rwanda, Sierra Leone, South Africa, Tanzania, Uganda, Zambia, and Zimbabwe) were included in the analysis. Exclusion criteria included guidelines not written in English and guidelines that did not include reference to ethical considerations related to involving persons with mental, cognitive, legal disability or dementia or Alzheimer’s disease in research. Ethics guidelines were analysed to explore degree of alignment with the CIOMS guidelines and principles of the CRPD. Guidelines that permitted the inclusion of participants with psychosocial disabilities in research were characterised by whether they approached inclusion from a starting point of safeguarding (in alignment with the CIOMS guidelines) or empowerment (in alignment with the CRPD). For example, where the sole requirement for inclusion of participants with psychosocial disabilities was the appointment of a surrogate decision-maker, the regulation was considered to adopt a safeguarding, rather than empowering, approach. Conversely, empowerment measures were those that sought to provide participants with some measure of control over their participation by providing for, among other things, advanced directives for consent to research or shared or supported decision-making models.

Ten of 14 guidelines reviewed offered conditions under which participation of persons with cognitive impairment is permissible. Similarly, 10 of 14 regulations also permitted proxy consent only from a legally authorised representative (LAR) or guardian. However, only four of these regulations provided guidance on who qualifies as LAR: Kenya (other appropriate representatives), Malawi (parents or legal guardians), Sierra Leone (responsible family member or LAR), South Africa (legally appropriate person: spouse or partner; parent; grandparent; adult child; brother or sister, according to the National Health Act 2002).

Only two countries—Liberia and South Africa—set out provisions recognizing advance consent or advance directives. In addition, only Sierra Leone and South Africa had provisions recognizing and upholding the participant’s refusal to participate as being superior to any proxy consent. Of the 14 countries whose documents were reviewed, those from Ethiopia, Ghana, Malawi, Rwanda, Uganda and Zimbabwe did not include any consideration of consent or assent by the participant.

### National case study 1: Malaysian Mental Health Act 2001

Although the CIOMS guidelines are regarded as one of the main references for research ethics governance in Malaysia, no formal guidance has been developed in the Malaysian context to adopt the recommendations by CIOMS to support the inclusion of people with psychosocial disabilities in research. There is some anecdotal evidence that the absence of specific guidance has in fact resulted in the exclusion of such individuals from participating in research ( Kaur, 2011), suggesting that this lack of formal guidance may adversely impact the inclusion of participants with psychosocial disabilities in research.

The sole regulatory instrument that addresses the recruitment of participants with psychosocial disabilities, even if only in brief, is the Malaysian *Mental Health Act 2001* (MHA). One section of the MHA, Section 77, provides guidance for people living with psychosocial disabilities to participate in clinical trials. The MHA states that if a potential participant is found to have capacity, the participant can provide consent by himself or herself. However, consent needs to be given by a relative if the potential participant is incapable of giving his or her own consent; in the event that there are no available relatives, two psychiatrists can provide consent on behalf of the potential participant.

This piece of legislation aligns more closely with a safeguarding approach, permitting surrogate decision-making after determination of incapacity. Significantly, paragraphs (1)(a) and (b) of Section 77 permit proxy decision-making with what appears to be unfettered decision-making powers; indeed, Section 77 makes no mention of the interests or will of the person at all. The MHA does not require the relatives or physicians of the person declared incompetent to even inquire about the person’s interests, much less consider the person’s interests in their decision. The MHA affords some degree of protection of the individual's interests in the context of clinical care (Section 86), but with no mention of research.

Malaysia’s MHA is insufficient to empower people with psychosocial disabilities to make their own decisions to participate in research.

### National case study 2: Peru’s Regulation on Clinical Trials

In 2018, Peru changed its legal framework on legal capacity and amended its Civil Code in accordance with the Article 12 of the CRPD. This new framework aims to recognize the legal capacity of people with disabilities on an equal basis by eliminating the figure of interdiction ^1^ and providing them support to exercise their legal capacity and make choices by themselves. Even though this is an important achievement towards equality in the country, it requires several modifications of other laws and regulations, including those related to health—including the Peruvian regulation on the conduct of clinical trials.

Peru’s clinical trials regulation was issued in 2017; the regulation has not yet been adapted to the Civil Code. In adherence with CIOMS guidelines, this regulation establishes that, to include participants with psychosocial disabilities who cannot decide by themselves, informed consent must be obtained by their legal representative. Given the evident legal conflict, two scenarios may be occurring in practice: 1) people with psychosocial disabilities are being systematically excluded from research, or 2) people living with psychosocial disabilities are being enrolled in research according to previous practice ( *i.e.*, with proxy consent). The latter scenario violates the CRPD, Peru’s Civil Code, and more generally, Peru’s constitutional order. In any case, both scenarios are ethically fraught: the first unfairly excludes people with psychosocial disabilities from research while the second does not treat them on an equal basis with others in accordance with the CRPD.

How to translate the Civil Code’s provisions on legal capacity into the clinical trials governance framework is a pending task in Peru. In 2021, the Ministry of Health, through the National Institute of Health, designated a Working Group to draft an update of Peru’s Regulation on Clinical Trials. Revising this regulation stands to offer the opportunity to discuss this specific topic in detail, engage relevant stakeholders, and refine principles and strategies to adapt the CRPD into the clinical trials regulations in order to ensure that clinical trials involving people with psychosocial disabilities are conducted ethically in Peru.

## Analysis

As made evident in our regional review of regulations to govern research in Africa, and through the in-depth case studies of regulations in Malaysia and Peru, the global regulatory landscape of research with people with psychosocial disabilities is varied. Most importantly, no regulations reviewed in this study appear to offer a clear harmonisation between the mandates of the CRPD and the recommendations of CIOMS with respect to research participation.

Our analysis of this review of regulations globally highlights a divide between ‘empowering’ versus ‘controlling’ or ‘safeguarding’ frameworks. In some country contexts, *e.g.* Liberia and South Africa, people living with psychosocial disabilities are able to create advance directives to govern decisions about their participation in research. Conversely, in some country contexts, *e.g.*, Malaysia, people with psychosocial disabilities can be enrolled in a research study on the basis of proxy consent. In some cases, as in Peru, disagreement between existing regulations creates a third ‘conflicting’ framework. Here, regulations must be streamlined to better accommodate the rights of persons living with psychosocial disabilities.

In each case, regulations may align more with the CRPD or with CIOMS. That said, no country analysed offered a fully CRPD-compliant approach to enabling research participation for people living with psychosocial disabilities. A core assertion of the CRPD, as further explicated in General Comment No. 1, is that legal capacity should not be determined by mental capacity ( UN, 2014)—an assertion that poses a clear challenge to standard research ethics regulations as prescribed by CIOMS. Indeed, while CIOMS concerns itself with capacity evaluations of potential participants as a strategy for ethical recruitment, the CRPD challenges the notion that ‘capacity’ can be objectively or meaningfully interpreted at all ( UN, 2008).

Moreover, the CRPD demands that people living with psychosocial disabilities are treated on an equal basis with others. This imperative challenges the common practice in research ethics of excluding potential participants on the basis of certain characteristics ( *e.g.*, disability).

In the next sections, we offer an analysis of how the CRPD and CIOMS could align. We then set forth a proposal for countries’ regulatory bodies to consider in revising their guidelines on the inclusion of people living with psychosocial disabilities in research.

### Fair research participation: CIOMS and the CRPD

We suggest that resolving tensions between the CRPD and CIOMS requires a closer analysis of rights with respect to research participation. CIOMS does not guarantee the ‘right to participate’ in research, though CIOMS arguably offers an implicit ‘right’ not to be unjustly excluded on the basis of a social category ( CIOMS, 2016). The CRPD also does not guarantee an explicit ‘right to participate’ in research ( UN, 2008), though some commentators have argued that the text of the Convention implies the right to participate in research ( Nilsson & Broström, 2020).

We argue that a more consistent alignment of the principles of the CRPD within the ethically stringent (and necessarily so) bounds of clinical research dictates that the CRPD implies a right to benefit from research as opposed to a right to *participate* in research, recognising that benefitting from research does not necessarily require participating in research. That is, while an overriding right to research participation should not be understood as a component of the ‘right to full participation in society’ as guaranteed in the CRPD, the right to benefit from research should be. We also suggest that the CRPD’s principles should apply to the research agenda as a whole, but not necessarily to research studies individually, drawing on Little *et al.*’s work on the inclusion of pregnant people in clinical research ( 2022).

### Proposal: aligning national research ethics regulations with CIOMS and the CRPD

Therefore, our proposal for aligning national research ethics regulations with both CIOMS and the CRPD turns on the view that the CRPD affords the right to *benefit* from research without affording an overriding right to participate in research.

Based on this understanding of fair research participation in the context of the CRPD, we suggest that the ethical application of core principles of the CRPD will depend on the *purpose* of the research. However, all research should subscribe to the CRPD-compliant view that the presence of psychosocial disability does not imply incapacity.

We put forth the following suggested revisions to national research ethics regulations:

- For research intended to be generalizable population-wide, individuals with psychosocial disabilities should be included as part of the general population. In these instances, researchers should respect the principle of non-discrimination by not excluding on the basis of psychosocial disability. However, this provision does not inherently afford a rights-based claim to participation in *any* ‘general population’ study ( *e.g.* a Phase II non-lifesaving vaccine efficacy trial); rather, this provision is intended to ensure accurate representation of the true population to maximize the validity of study findings ( *e.g.* an implementation study for a flu vaccine rollout).
- For research intended to benefit a specific group of individuals living with psychosocial disabilities, and therefore recruiting only people living with psychosocial disabilities, researchers should ensure that assessments of capacity to consent to research are not based on disability, but rather on general principles for informed consent in research. If the person does not consent to research participation, researchers should not, under any circumstance, accept proxy consent to participate.
- All research should accept that the presence of psychosocial disability does not imply incapacity.

This proposal, we contend, attends to both ethical imperatives of equal treatment and universal legal capacity, as set forth by the CRPD, while ensuring that standard research ethics regulations, as set out in CIOMS, are met.

## Data Availability

No data are associated with this article. The authors thank Ms Adrienne Hunt, Global Forum on Bioethics in Research Secretariat, for support throughout preparation of this manuscript. The authors are grateful to attendees of the GFBR 2021 for their contributions to discussion on this important topic during the conference. **Marisha Wickremsinhe** London School of Hygiene and Tropical Medicine, UK *Roles*: Conceptualization, Writing - Original Draft Preparation, Writing - Review Editing **Sarah Carracedo** Pontifical Catholic University of Peru *Roles*: Conceptualization, Writing - Original Draft Preparation, Writing - Review Editing Email address: sarah.carracedo@pucp.edu.pe **Aminu Yakubu** University of Ibadan, Nigeria Center for Bioethics and Research, Ibadan, Nigeria *Roles*: Conceptualization, Writing - Original Draft Preparation, Writing - Review Editing Email address: yakubu.aminu@gmail.com **Aimi Nadia Mohd Yusof** Universiti Teknologi MARA, Malaysia *Roles*: Conceptualization, Writing - Original Draft Preparation, Writing - Review Editing Email address: aiminadia@uitm.edu.my **Sharon Kaur** Universiti Malaya, Malaysia *Roles*: Conceptualization, Supervision, Writing - Original Draft Preparation, Writing - Review Editing Email address: kaursh@um.edu.my

## Footnotes

^1^ “Interdiction” is a process in which a court appoints a LAR to a person with mental impairments after a capacity assessment that determines if they *can* decide by themself, and *to what extent (*Art. 564, Peruvian Civil Code, 1984).
