# Supplementary material for: Governance of research involving people with psychosocial disabilities
Source: Wellcome Open Res. 2023 Jun 2;8:231. [Version 1] doi: 10.12688/wellcomeopenres.19279.1 (PMC11358677; doi:10.12688/wellcomeopenres.19279.1)
Supplement: Peer review history

# Reviewer report: Reviewer response for version 1

**DOI:** 10.21956/wellcomeopenres.21362.r66872

**Published:** 2024-08-28

**Authors:** Alexandra Devine

**Affiliations:** 1 Melbourne School of Population and Global Health, The University of Melbourne, Melbourne, Victoria, Australia

Thank you for the opportunity to review this important yet complex analysis examining the tensions and alignment between the UNCRPD and CIOMS in relation to the ethical inclusion of people with psychosocial disability within research and their right to benefit from ethical research on an equal basis with others.

 Given the complexity of the topic, I concur with earlier reviews that it would be useful to strengthen the background and rationale for the analysis. I add to this a suggestion to include the reasons or benefits for the selection of the three jurisdictions examined. What can each jurisdiction learn from each other?   

 The paper highlights the question as to whether a clear harmonization between the UNCRPD and CIOMS is currently possible? Or are revisions of the CIOMS to better align with the UNCRPD called for? Within this, should the emphasis be on the right to choose whether to participate in research on an equal basis with others (as opposed to substituted decision-making), rather than trying to delineate between the right to participate in research versus the right to benefit from research? In agreeance with the outlined provisions within the protocols of Sierra Leone and South Africa to uphold a potential participant’s refusal to participate in research as being superior to any proxy consent, I concur with your suggestion that CIOMS and other ethical guidelines should move away from substituted decision-making, towards clearer guidance on supported decision-making (where required and with the clear assent of potential research participants) incorporated within strengthened capacity to provide informed consent processed with all potential research participants, regardless of the presence or absence of a diagnosed or undiagnosed condition. Could your first recommendation in relation to generalizable population-based research also reiterate the above point? Simultaneously, are there opportunities within the context of the UNCRPD to strengthen its guidance on the ethical inclusion of people with disabilities within research, beyond the right to benefit from research?

Does the article adequately reference differing views and opinions?

Partly

Are all factual statements correct, and are statements and arguments made adequately supported by citations?

Partly

Is the Open Letter written in accessible language?

Yes

Where applicable, are recommendations and next steps explained clearly for others to follow?

Partly

Is the rationale for the Open Letter provided in sufficient detail?

Partly

Reviewer Expertise:

I am predominantly a qualitative researcher focusing on inclusion of people with disabilities within society and systems.

I confirm that I have read this submission and believe that I have an appropriate level of expertise to confirm that it is of an acceptable scientific standard.

# Reviewer report: Reviewer response for version 1

**DOI:** 10.21956/wellcomeopenres.21362.r66867

**Published:** 2023-10-13

**Authors:** Cristian R Montenegro

**Affiliations:** 1 Wellcome Centre for Cultures and Environments of Health, University of Exeter, Exeter, England, UK

Thanks for the opportunity to comment on this insightful and important letter. While the CRPD has significantly influenced advocacy and normative fields, its actual and potential impact on research ethics remains underexplored. This paper usefully addresses this gap by contrasting the CRPD with the CIOMS guidelines.

 My only concern relates to the connection between the first and second parts of the letter—specifically, the transition from reviewing selected regulations to contrasting the CIOMS guidelines and the CRPD. While you argue that the review reveals tensions between the two frameworks, it's not clear how this empirical examination of country cases directly informs the proposal you develop at the end. Your proposal to reconcile the tensions between the CRPD and CIOMS based on the notion of a "right to benefit from research" is well-justified. However, the paper could be strengthened by elaborating on how this proposal might address the regulatory complexities in the countries you've reviewed, even if this is in speculative terms.

Does the article adequately reference differing views and opinions?

Yes

Are all factual statements correct, and are statements and arguments made adequately supported by citations?

Yes

Is the Open Letter written in accessible language?

Yes

Where applicable, are recommendations and next steps explained clearly for others to follow?

Partly

Is the rationale for the Open Letter provided in sufficient detail?

Partly

Reviewer Expertise:

South America; Mental Health Policy; Global Mental Health; Human Rights and Mental Health; Qualitative Research; Social Theory and Medicine

I confirm that I have read this submission and believe that I have an appropriate level of expertise to confirm that it is of an acceptable scientific standard, however I have significant reservations, as outlined above.

# Reviewer report: Reviewer response for version 1

**DOI:** 10.21956/wellcomeopenres.21362.r66864

**Published:** 2023-10-12

**Authors:** Sharon Kleintjes

**Affiliations:** 1 Department of Psychiatry and Mental Health, University of Cape Town, Rondebosch, Western Cape, South Africa

This open letter addresses is a useful contribution to consideration of ethical conduct of research with people with psychosocial disabilities.

**Is the rationale for the Open Letter provided in sufficient detail?**

 Yes

**Does the article adequately reference differing views and opinions?**

 Yes

 Comment: Within the scope of the paper which the authors have set for their enquiry.

**Are all factual statements correct, and are statements and arguments made adequately supported by citations?**

 Partly

 Comment regarding the statement “Ten of 14 guidelines reviewed offered conditions under which participation of persons with cognitive impairment is permissible": The article focuses on persons with psychosocial disabilities. I recommend that the term 'psychosocial disability' be used throughout, rather than using this term interchangeably with 'cognitive impairment'. People with psychosocial disabilities are those who have disabling impacts of experiencing a mental disorder or illness, which in some instances or at points of acute illness, or due to medication and other treatment effects may include cognitive impairment (attentional, concentration problems) but psychosocial disabilities are not synonymous with cognitive impairment, nor is the latter experienced for the most part by all people with psychosocial disability all of the time, as may be the case with person with significant cognitive impairment, for example, a person diagnosed with intellectual disability, or dementia. Persons with cognitive impairment may have a concurrent/co-occurring psychosocial disability, for example a person with an intellectual disability also having depression or schizophrenia. It may also be useful for the authors to define what they mean by “psychosocial disability” See panel 4 of the Lancet Commission on Global Mental Health ^1^.

 The authors note that “We also suggest that the CRPD’s principles should apply to the research agenda as a whole, but not necessarily to research studies individually, drawing on Little *et al.*’s work on the inclusion of pregnant people in clinical research ( 2022)”. The authors are encouraged to note what specific elements of this work is relevant to their paper, given that their argument in the next section of the paper seems in part to rest on these findings and conclusions. The article is a book chapter, which will not readily be accessible to all readers of the paper.

**Is the Open Letter written in accessible language?**

 Yes

**Where applicable, are recommendations and next steps explained clearly for others to follow?**

 Partly

 Additional discussion of the recommendations/suggestions would assist to locate the recommendations in the context of the complex social milieus within which research ta with people with psychosocial disabilities takes place. The authors note, for example, that recruitment of people living with psychosocial disabilities should ensure that assessments of capacity to consent to research are not based on disability, but rather on general principles for informed consent in research. While capacity to consent should not be based on disability, researchers, in seeking informed consent, should include consideration of other intersecting issues people with psychosocial disability encounter which might arise during research which are not a consideration for people without this disability, for example, stigma, discrimination, social exclusion and potential for harm when identified as mentally ill in some settings during research, the poverty-mental health link and possible pressure to participate for financial gain, and the intersection of other vulnerabilities identified in guideline 15-17 of the CIOMS ^2^.

 The authors note that their “proposal for aligning national research ethics regulations with both CIOMS and the CRPD turns on the view that the CRPD affords the right to benefit from research without affording an overriding right to participate in research. The CRPD does not demand “an overriding right to participate in research”, but as the authors note, some writers note that participation in research may be implied. Certainly the CRPD does demands that people living with psychosocial disabilities are treated on an equal basis with others, that is, that as others without disabilities are included, so should people with psychosocial disabilities be included. Given that people with psychosocial disability have historically been excluded from research, and that their participation is still overlooked in many instances, care still needs to be taken to note the need for attention to and enablement of their participation in research which impacts on their lives, when making any statement about their right - or the nature of that right - to participate in research, so as to avoid any inadvertent misreading that it is implied that they may continue to be left out of relevant research.

Does the article adequately reference differing views and opinions?

Yes

Are all factual statements correct, and are statements and arguments made adequately supported by citations?

Partly

Is the Open Letter written in accessible language?

Yes

Where applicable, are recommendations and next steps explained clearly for others to follow?

Partly

Is the rationale for the Open Letter provided in sufficient detail?

Yes

Reviewer Expertise:

Mental health policy and service development, participation of people with psychosocial disability in MH policy and service development. self-advocacy by people with intellectual disability

I confirm that I have read this submission and believe that I have an appropriate level of expertise to confirm that it is of an acceptable scientific standard, however I have significant reservations, as outlined above.

## References

[1] : The Lancet Commission on global mental health and sustainable development. *Lancet* .2018;392(10157) : 10.1016/S0140-6736(18)31612-X 1553-1598 10.1016/S0140-6736(18)31612-X 30314863

[2] : The Health Stigma and Discrimination Framework: a global, crosscutting framework to inform research, intervention development, and policy on health-related stigmas. *BMC Medicine* .2019;17(1) : 10.1186/s12916-019-1271-3 10.1186/s12916-019-1271-3 30764826 PMC6376797

# Reviewer report: Reviewer response for version 1

**DOI:** 10.21956/wellcomeopenres.21362.r66873

**Published:** 2023-10-05

**Authors:** Ikenna Ebuenyi

**Affiliations:** 1 Department of Rehabilitation Science and Technology, University of Pittsburgh, Pittsburgh, Pennsylvania, USA

Thank you for inviting me to review this very important letter. The subject is relevant and examines critical issues related to informed consent, inclusion, and equal participation of persons with psychosocial disabilities in research. I have a few comments and suggestions that should strengthen the discourse.

1. It is essential that the authors further clarify the difference between appointment of a ‘surrogate decision maker’ and ‘advanced directives or supported decision-making models’. The authors have classified the former as a safeguarding measure (associated with CIOM) and the latter as empowering and allowing some measure of control (associated with CRPD). The difference between viewpoints is not sufficiently explored and it is pertinent to highlight that ‘some measure of control’ may not align with the absolute stance of the CRPD on legal capacity.
2. Although the authors describe their eligibility criteria for the included African countries, it is not evident if these countries have signed or ratified the CRPD. A few sentences or table on this will provide important legal context regarding the compliance of their national laws to the CRPD.
3. The case studies about Peru and Malaysia are interesting; however, the rationale for choosing both counties are missing. The Malaysia Mental Health Act 2001 (MHA) described by the authors predates the CRPD and expectedly, it does not align with the CRPD. If the case studies are there to further illustrate relevance of the CIOMs or CRPD mandate, would it not be better to use countries with legislations enacted after these instruments? Again, this may be the possible explanation for Liberia having a national policy/regulation that aligns with the CRPD or the ‘empowering framework’.
4. In conclusion, I consider the recommendations in the letter very interesting and useful. They would elicit further conversations and questions. For instance, the authors recommendation that ‘If the person does not consent to research participation, researchers should not, under any circumstance, accept proxy consent to participate’ poses the question:  does it include or preclude advance directives?

Does the article adequately reference differing views and opinions?

Partly

Are all factual statements correct, and are statements and arguments made adequately supported by citations?

Yes

Is the Open Letter written in accessible language?

Yes

Where applicable, are recommendations and next steps explained clearly for others to follow?

Partly

Is the rationale for the Open Letter provided in sufficient detail?

Partly

Reviewer Expertise:

Psychosocial Rehabilitation, Disability inclusion and Health Policy & Systems

I confirm that I have read this submission and believe that I have an appropriate level of expertise to confirm that it is of an acceptable scientific standard, however I have significant reservations, as outlined above.

# Reviewer report: Reviewer response for version 1

**DOI:** 10.21956/wellcomeopenres.21362.r66871

**Published:** 2023-09-27

**Authors:** Lana Van Niekerk

**Affiliations:** 1 Department of Occupational Therap​y, Stellenbosch University, Stellenbosch, Western Cape, South Africa

The letter foregrounds an important consideration for researchers in general and those who seek to explore issues specifically pertaining people with psychosocial disabilities. The current tendency to exclude persons with psychosocial disability based on assumptions of incapacity is indeed problematic, exclusionary, and counterproductive. The authors' use of the CRDP and CIOMS as a foundation for analysis ensures the relevance and application value of recommendations made in the letter. 

 I was left wondering about the connection between the two parts of the letter. It would improve the letter if the linkages between the first part, in which guidelines from African countries were 'characterised', are clarified. While the focus on African countries is appreciated as a welcome change from the usual omission of these countries, the rationale for focussing exclusively on African countries was not provided. A sentence of two to improve the link between the two parts of the letter will improve the coherence.

 The authors set out to "characterise the nature of a sample of existing ethics guidelines". In my mind, this would entail a full exploration of the nature of the guidelines included in the sample. However, the letter comments on two very specific aspects. I suggest the authors' stated intention should be clarified to focus the reader more accurately on the elements extracted from the ethics guidelines.

Does the article adequately reference differing views and opinions?

Yes

Are all factual statements correct, and are statements and arguments made adequately supported by citations?

Partly

Is the Open Letter written in accessible language?

Yes

Where applicable, are recommendations and next steps explained clearly for others to follow?

Yes

Is the rationale for the Open Letter provided in sufficient detail?

Yes

Reviewer Expertise:

Disability and Work; Occupational Science; Occupational Therapy; Community Mobility.

I confirm that I have read this submission and believe that I have an appropriate level of expertise to confirm that it is of an acceptable scientific standard, however I have significant reservations, as outlined above.

# Reviewer report: Reviewer response for version 1

**DOI:** 10.21956/wellcomeopenres.21362.r61474

**Published:** 2023-07-24

**Authors:** Kiran Manku

**Affiliations:** 1 Department of Clinical Medicine, University of Oxford, Oxford, England, UK

The letter makes an interesting contribution to the literature, as it lays out the critical issue of a lack of consistent guidance on the inclusion of persons with psychosocial disabilities in research. 

 I feel that the letter could be strengthened with clarifications regarding the rationale, interpretation of existing guidance, and the proposal they make, as outlined below.

 Specifically, in the introduction it would be useful to have more detail about the origins and intentions of the CIOMS and CRPD. I think it might be helpful to highlight that the CRPD was made with persons with disabilities. In addition, clarification on the CIOMS as a means to ensure a fair distribution of research benefits and the interpretations of safeguarding and “controlling” approaches could be explained in the introduction. 

 For example, it might be useful to preface the introduction about CIOMS and the CRPD, with an overview of inclusion of people with psychosocial disabilities in research. This would help lay out how they were developed and the concepts of inclusion for scientific validity, “safeguarding”, “controlling”, and “empowerment”. I think these concepts should be clarified for the audience to understand the different perspectives of inclusion in research.

 I found the review and analysis very interesting. Across the countries included, I know of local advocacy groups for persons with psychosocial disabilities who advocate for the right to decide, including in research participation. Although their work isn’t within the remit of this analysis and review, I wonder if their work could be drawn upon within country settings, should a country want to engage in adopting or creating governance for research including persons with psychosocial disabilities.

 The proposed suggestion is helpful but could include some good examples. It would be useful to have templates of assent or measures of capacity where research is only recruiting people with psychosocial disabilities. I also wonder if the paper should state that it would be good practice to include persons with psychosocial disabilities in co-creating these consent tools (and the wider research itself). 

 Overall, I think the letter highlights an issue that needs more attention and could be clarified to make the case clearer.

Does the article adequately reference differing views and opinions?

Partly

Are all factual statements correct, and are statements and arguments made adequately supported by citations?

Yes

Is the Open Letter written in accessible language?

Yes

Where applicable, are recommendations and next steps explained clearly for others to follow?

Partly

Is the rationale for the Open Letter provided in sufficient detail?

Partly

Reviewer Expertise:

Disability, inclusion, mental health, bioethics, research ethics.

I confirm that I have read this submission and believe that I have an appropriate level of expertise to confirm that it is of an acceptable scientific standard, however I have significant reservations, as outlined above.
